# Personalized prediction of breast cancer candidates for Anti-HER2 therapy using ^18^F-FDG PET/CT parameters and machine learning: a dual-center study

**DOI:** 10.3389/fonc.2025.1590769

**Published:** 2025-05-14

**Authors:** Zhenguo Sun, Jianxiong Gao, Wenji Yu, Xiaoshuai Yuan, Peng Du, Peng Chen, Yuetao Wang

**Affiliations:** ^1^ Department of Nuclear Medicine, The Third Affiliated Hospital of Soochow University, Changzhou, Jiangsu, China; ^2^ Department of Nuclear Medicine, The First People’s Hospital of Lianyungang/The First Affiliated Hospital of Kangda College of Nanjing Medical University, Lianyungang, Jiangsu, China

**Keywords:** breast cancer, 18 F-FDG PET/CT, HER2, machine learning, SHAP

## Abstract

**Background:**

Accurately evaluating human epidermal growth factor receptor (HER2) expression status in breast cancer enables clinicians to develop individualized treatment plans and improve patient prognosis. The purpose of this study was to assess the performance of a machine learning (ML) model that was developed using ^18^F-FDG PET/CT parameters and clinicopathological features in distinguishing different levels of HER2 expression in breast cancer.

**Methods:**

This retrospective study enrolled breast cancer patients who underwent ^18^F-FDG PET/CT scans prior to treatment at Lianyungang First People’s Hospital (centre 1, n=157) and the Third Affiliated Hospital of Soochow University (centre 2, n=84). Two classification tasks were analysed: distinguishing HER2-zero expression from HER2-low/positive expression (Task 1) and distinguishing HER2-low expression from HER2-positive expression (Task 2). For each task, patients from Centre 1 were randomly divided into training and internal test sets at a 7:3 ratio, whereas patients from Centre 2 served as an external test set. The prediction models included logistic regression (LR), support vector machine (SVM), extreme gradient boosting (XGBoost) and multilayer perceptron (MLP), and SHAP analysis provided model interpretability. Model performance was evaluated via the area under the receiver operating characteristic curve (AUC), accuracy, sensitivity, specificity, positive predictive value (PPV) and negative predictive value (NPV).

**Results:**

XGBoost models exhibited the best predictive performance in both tasks. For Task 1, recursive feature elimination (RFE) was used to select 8 features, excluding pathological features, and the XGBoost model achieved AUCs of 0.888, 0.844 and 0.759 for the training, internal and external testing sets, respectively. The top three features according to the SHAP values were the tumour minimum diameter, mean standardized uptake value (SUVmean) and CTmean. For Task 2, 9 features were selected, including progesterone receptor (PR) status as a pathological feature. The XGBoost model achieved AUCs of 0.920, 0.814 and 0.693 for the training, internal and external testing sets, respectively. The top three features according to the SHAP values were the PR status, maximum tumour diameter and metabolic tumour volume (MTV).

**Conclusions:**

ML models that incorporate ^18^F-FDG PET/CT parameters and clinicopathological features can aid in the prediction of different HER2 expression statuses in breast cancer.

## 1 Introduction

In China, breast cancer is the second most commonly diagnosed malignancy and the fifth leading cause of cancer-related death among women (1). Human epidermal growth factor receptor 2 (HER2), which is a member of the tyrosine kinase receptor family, plays a pivotal role in regulating cell growth, survival and metastatic progression. HER2 overexpression is observed in approximately 20–30% of breast cancer cases (2, 3). According to the 2018 American Society of Clinical Oncology (ASCO) and College of American Pathologists (CAP) guidelines, HER2 overexpression is identified on the basis of immunohistochemistry (IHC) and *in situ* hybridization (ISH) results. Specifically, positive HER2 expression is defined as an IHC score of 3+ or an IHC score of 2+ with ISH amplification, whereas negative HER2 expression is defined as an IHC score of 0+ or 1+ or an IHC score of 2+ without ISH amplification (4). Traditionally, the most reliable predictive factor for determining the likelihood of patient response to anti-HER2 agents is HER2 overexpression or amplification. Consequently, only patients with HER2-positive disease receive anti-HER2 drug therapy (3, 5, 6). Currently, the National Comprehensive Cancer Network (NCCN) and ASCO guidelines recommend chemotherapy combined with trastuzumab as neoadjuvant therapy for early-stage HER2-positive breast cancer, with the aim of reducing the tumour burden and optimizing surgical outcomes (7, 8). Therefore, preoperative determination of the HER2 expression status of breast cancer has significant clinical value.

In 2023, the European Society for Medical Oncology (ESMO) defined HER2-low breast cancer as tumours with a HER2 IHC score of 1+ or 2+ without ISH amplification (9). HER2-low breast cancer accounts for more than half of all traditional HER2-negative breast cancers. Compared with HER2-zero or HER2-positive breast cancers, HER2-low breast cancer has distinct biological characteristics and clinical prognoses (3, 10, 11). Recent clinical trials have demonstrated that patients with HER2-positive breast cancer as well as patients with HER2-low breast cancer exhibit high response rates to HER2-targeted antibody drug conjugates, such as trastuzumab (DS-8201) (12, 13). Notably, the phase III DESTINY-Breast04 (DB-04) trial has shown that trastuzumab deruxtecan significantly improves overall survival compared with conventional chemotherapy in patients with pretreated HER2-low metastatic breast cancer (14). Consequently, identifying this specific subgroup of breast cancer may optimize the strategy for treating traditional HER2-negative breast cancer.

The preoperative HER2 status of breast cancer is primarily determined via analysis of percutaneous biopsy samples by IHC and ISH (15). However, owing to tumour heterogeneity, a single biopsy sample may not always be representative of the entire tumour (16). Moreover, the literature reports that incorporating the HER2-low category into the assessment of HER2 status can decrease the consistency of results obtained from core needle biopsy (CNB) and surgical resection specimens (17). The phenomenon by which a subset of tumours that were initially classified as HER2-zero via CNB are reclassified as HER2-low via surgical resection samples can be attributed to limitations that are inherent to the current semiquantitative HER2 IHC scoring system. Notably, this scoring system was originally designed to identify HER2-positive populations, resulting in subjective distinctions between HER2 IHC 0 and 1+ scores that are susceptible to interobserver variability (17, 18). In particular, achieving consistent interpretation of IHC 0 versus 1+ scores remains a critical challenge in the accurate diagnosis of HER2-low status (19).

Additionally, equivocal or critical IHC results, such as HER2 IHC 2+, are observed in approximately 15–20% of breast cancer cases (20). Even with known IHC results, HER2 IHC 2+ patients still require further ISH testing to identify HER2-low breast cancer. However, ISH testing is costly and time-consuming, and it demands stringent quality control. Therefore, there is an urgent need for new tools to accurately evaluate the HER2 expression status of patients with breast cancer in order to more quickly and accurately develop individualized treatment plans and to improve the prognosis of patients with breast cancer.

Plasma carcinoembryonic antigen (CEA), cancer antigen 125 (CA125) and cancer antigen 15-3 (CA15-3) are among the tumour markers that are most commonly used in the diagnosis of breast cancer (21). Previous studies have indicated that the serum levels of CEA and CA15-3 may vary across different molecular subtypes of breast cancer, and the preoperative levels of CEA and CA15-3 have been shown to significantly impact the prognosis of Chinese women with breast cancer (22, 23). However, the role of these commonly used serum tumour markers in predicting HER2 expression status in breast cancer remains a subject of ongoing debate.

^18^F-FDG PET/CT is a noninvasive molecular imaging technique that can provide comprehensive information about tumour metabolism. Key metabolic parameters derived from PET/CT, including the maximum standardized uptake value (SUVmax) and metabolic tumour volume (MTV), enable a more precise assessment of tumour heterogeneity and serve as valuable biomarkers for tailoring therapeutic strategies (24, 25). Studies by Gao et al. and Gui et al. have demonstrated that the SUVmax is correlated with HER2 expression status, with HER2-positive tumours exhibiting higher SUVmax values (26, 27). However, previous studies have not integrated multiparameter PET/CT features to develop predictive models.

Recent advances in artificial intelligence and machine learning (ML) have revolutionized oncological imaging, particularly in the areas of key feature extraction and model development (28–30). Although ML models based on MR imaging features have been validated for differentiating HER2 expression states (31, 32), the potential of PET/CT multiparametric and clinicopathological features remains unexplored. Therefore, this study aimed to develop and validate multiple ML models using pretreatment ^18^F-FDG PET/CT parameters and clinicopathological features to evaluate the HER2 expression status of breast cancer patients. Leveraging dual-centre datasets for robust validation, we further employed SHAP analysis to provide both population-level feature importance rankings and individualized prediction visualizations, thus improving the clinical interpretability of multiparametric decision-making processes.

## 2 Materials and methods

### 2.1 Study population

This retrospective study enrolled breast cancer patients who underwent 18F-FDG PET/CT examinations before treatment at two centres; patients were enrolled from Lianyungang First People’s Hospital (Centre 1) between October 2017 and March 2024 and from the Third Affiliated Hospital of Soochow University (Centre 2) between January 2013 and March 2024. The inclusion criteria were as follows: (1) pathologically confirmed unilateral primary breast cancer, with pathological results derived from surgical resection or biopsy; (2) no more than 30 days between the completion of the ^18^F-FDG PET/CT examination and the surgery or biopsy; (3) no prior treatments, such as surgery, endocrine therapy, radiotherapy, or chemotherapy, before the ^18^F-FDG PET/CT examination; (4) clearly defined HER2 test results; and (5) no history of other malignant tumours. The exclusion criteria were as follows: (1) the presence of other breast diseases that could interfere with breast cancer imaging concurrently; (2) poor quality of PET/CT images due to artefacts or other factors; and (3) incomplete clinical data or immunohistochemical information.

**Figure 1:**
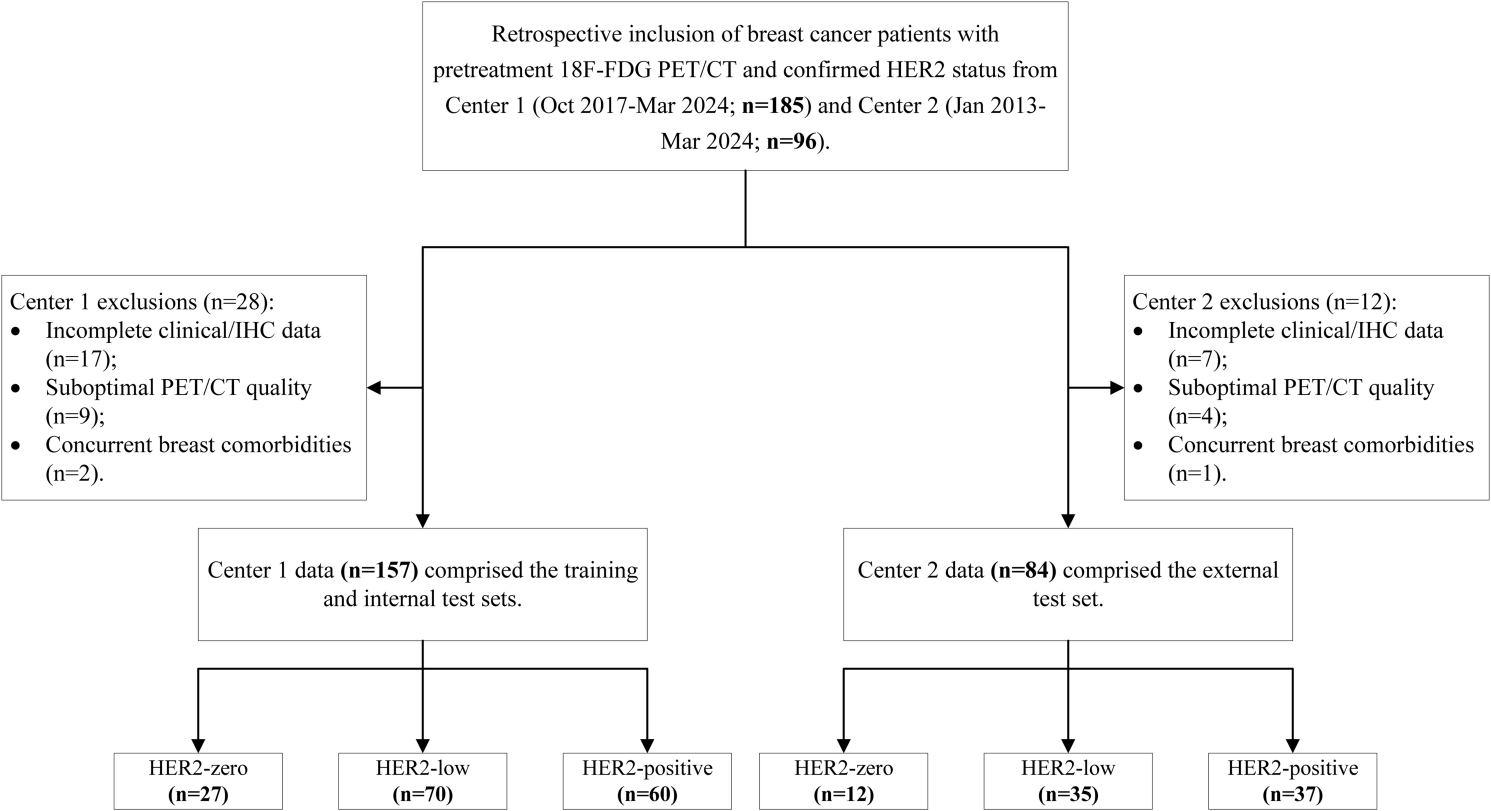
Patient enrolment pathway at the two institutions.

A total of 241 breast cancer patients (157 patients from Centre 1 and 84 patients from Centre 2) were enrolled on the basis of the aforementioned criteria. All the patients were divided into three groups on the basis of HER2 expression status: the HER2-zero, HER2-low and HER2-positive groups. Clinical pathological information was obtained through the retrieval of medical records and included data about age, tumour marker levels, oestrogen receptor (ER) status, progesterone receptor (PR) status, Ki67 index, menopausal status, lymph node metastasis and distant metastasis. The process of study population enrolment is shown in **Figure 1**.

### 2.2 Image acquisition and analysis

Image acquisition was performed using Siemens Biograph mCT flow 64 PET/CT scanners at both hospitals. All the patients fasted for 4–6 hours before the examination. Patient weight, height, and fasting blood glucose levels were recorded on the day of examination. Patients were intravenously injected with ^18^F-FDG, with a radiochemical purity >95% and a standard dose of 3.70–5.55 MBq/kg. Imaging was conducted 60 minutes postinjection. Patients were placed in the supine position for both the CT and PET scans. The respiratory gating mode was used with a speed of 1.5 mm/s and a matrix of 200×200. The PET/CT imaging range extended from the skull base to the mid-thigh. Images were reconstructed using the UltraHD iterative method, producing transverse, sagittal, and coronal sections along with fusion images.

Two physicians with 3 years of experience in nuclear medicine imaging diagnosis utilized 3D Slicer software (version 4.11.2, http://www.slicer.org) to perform semiautomatic segmentation of regions of interest (ROIs) on the PET images. For the CT images, the ROIs were manually delineated layer by layer. All the completed ROIs were reviewed and validated by a senior nuclear medicine physician with over 20 years of PET/CT diagnostic experience. The parameters that were analysed on the basis of the delineated ROIs included the tumour max diameter, tumour min diameter, SUVmax, mean standardized uptake value (SUVmean), peak standardized uptake value (SUVpeak), MTV and total lesion glycolysis (TLG, which is the product of the MTV and SUVmean). The tumour max diameter refers to the longest dimension measured on the maximum cross-sectional CT image of the lesion, whereas the tumour min diameter is the maximum perpendicular measurement taken within the same plane and orthogonal to the long axis.

### 2.3 Feature selection and machine learning modelling

First, models to differentiate between HER2-zero and HER2-low/positive tumours (Task 1) were constructed. The complete dataset from Centre 1 was randomly stratified into a training set and an internal test set at a 7:3 ratio, while the complete dataset from Centre 2 was used as an external dataset. To avoid model overfitting, recursive feature elimination (RFE) was applied to the standardized data to select the optimal feature set. This method ensures the fairness of feature weight evaluation by eliminating the differences in dimensions and simultaneously selects the subset of features with the highest discriminative power for the target variable. Standardization preprocessing prevents high-variance features from dominating model training and enhances the stability of the RFE feature ranking. RFE, on the other hand, optimizes model complexity and generalization ability by recursively eliminating redundant features. The combined effect of these two methods effectively reduces the risk of overfitting and increases the interpretability of the model.

ML models were built using logistic regression (LR), support vector machine (SVM), extreme gradient boosting (XGBoost) and multilayer perceptron (MLP) algorithms from the Sklearn (version 1.3.2, https://scikit-learn.org/) module on the basis of the selected optimal feature set. Grid search and 5-fold cross-validation were used on the training set to find the best model parameters, and the model was then refit to the training set. During the model training process, the parameter class_weight was set to “balanced”, which dynamically adjusts the class weights. This approach allows the model to more effectively learn features from minority classes, thereby to some extent mitigating prediction bias issues caused by class imbalance in the data.

Additionally, a separate dataset of 202 HER2-low/positive patients was extracted to build models to differentiate between HER2-low and HER2-positive patients (Task 2). This group included 70 HER2-low and 60 HER2-positive patients from Centre 1 and 35 HER2-low and 37 HER2-positive patients from Centre 2. The extracted dataset from Centre 1 was again randomly stratified into a training set and an internal test set at a 7:3 ratio, while the extracted data from Centre 2 were used as an external dataset. The same feature selection method and ML model construction approach were applied to these data.

### 2.4 Statistical methods

R software (version 3.4.3, http://R-project.org/) was used to perform the statistical analyses. Continuous variables are presented as the means ± standard deviations for normally distributed data or as the medians (Q1–Q3) for skewed distributions. Categorical variables are presented as frequencies or percentages. Chi-square tests (categorical variables), t tests (normal distribution), or Mann–Whitney U tests (skewed distribution) were used to detect differences in clinicopathological and PET/CT imaging features among patients with different HER2 expression statuses. We evaluated the model’s performance using the receiver operating characteristic (ROC) curve and the area under the curve (AUC), we calculated metrics such as accuracy, sensitivity, specificity, positive predictive value (PPV) and negative predictive value (NPV), and decision curve analysis (DCA) was applied to assess the net clinical benefit of the models. The SHAP module was used to interpret the best-performing model, providing a visual representation of feature importance and facilitating personalized predictions. Pairwise comparisons of the AUCs of the models were conducted using DeLong’s test. A two-sided P<0.05 was considered to indicate statistical significance.

## 3 Results

### 3.1 General clinical characteristics of the patients

A total of 241 patients were included in the study; 157 patients were from Centre 1, and 84 patients were from Centre 2. Patients were divided into three groups according to their HER2 expression status: HER2-zero (39 patients, 16.18%), HER2-low (105 patients, 43.57%) and HER2-positive (97 patients, 40.25%). A comparison of the clinical, pathological and PET/CT parameters of the patients in these groups is detailed in **Tables 1**, **2**. There were significant differences (P < 0.05) in CA125 levels, maximum tumour diameters, minimum tumour diameters, SUVmax, SUVmean, SUVpeak, ER status and PR status across the different HER2 expression status groups.

**Table 1:** Clinicopathological features of patients with different HER2 expression statuses.

Variables	HER2-zero (n=39)	HER2-low (n=105)	HER2-positive (n=97)	P	P^1^	P^2^	P^3^
**Age**	59.82 ± 13.00	56.54 ± 12.50	55.77 ± 10.44	0.191	0.169	0.074	0.504
**CEA (ng/ml)**	2.26 (1.32-3.89)	1.88 (1.34-3.24)	2.21 (1.19-3.62)	0.788	0.659	0.597	0.766
**CA125 (ng/ml)**	13.90 (10.04-23.38)	12.10 (8.96-19.04)	12.40 (7.94-19.70)	**0.015**	**0.042**	0.241	0.553
**CA153 (ng/ml)**	12.63 (9.02-22.23)	14.60 (8.98-20.80)	11.62 (8.44-21.00)	0.489	0.527	0.501	0.274
Menopause				0.515	0.477	0.256	0.551
Negative	27 (69.23%)	66 (62.86%)	57 (58.76%)				
Positive	12 (30.77%)	39 (37.14%)	40 (41.24%)				
Lymph Node Metastasis				0.072	0.625	0.043	0.052
Negative	12 (30.77%)	28 (26.67%)	15 (15.46%)				
Positive	27 (69.23%)	77 (73.33%)	82 (84.54%)				
Distant Metastasis				0.354	0.208	0.152	0.780
Negative	35 (89.74%)	85 (80.95%)	77 (79.38%)				
Positive	4 (10.26%)	20 (19.05%)	20 (20.62%)				
ER status				<0.001	0.124	0.025	<0.001
Negative	13 (33.33%)	22 (20.95%)	53 (54.64%)				
Positive	26 (66.67%)	83 (79.05%)	44 (45.36%)				
PR status				<0.001	0.028	0.051	<0.001
Negative	20 (51.28%)	33 (31.43%)	67 (69.07%)				
Positive	19 (48.72%)	72 (68.57%)	30 (30.93%)				
Ki67 index				0.315	0.480	0.671	0.131
Negative	16 (41.03%)	50 (47.62%)	36 (37.11%)				
Positive	23 (58.97%)	55 (52.38%)	61 (62.89%)				

HER2, human epidermal receptor 2; ER, estrogen receptor; PR, progesterone receptor; p^1^, HER2-zero group vs. HER2-low group; p^2^, HER2-zero group vs. HER2-positive group; p^3^, HER2-low group vs. HER2-positive group.

The bold values indicate that the P-values are less than 0.05, which means they are statistically significant.

**Table 2:** PET/CT parameters of patients with different HER2 expression statuses.

Variables	HER2-zero (n=39)	HER2-low (n=105)	HER2-positive (n=97)	P	P^1^	P^2^	P^3^
**CTmax**	72.00 (64.00-82.50)	74.00 (65.00-82.00)	74.00 (65.00-84.00)	0.277	0.781	0.352	0.721
**CTmin**	18.27 (7.87-25.05)	20.22 (12.42-26.12)	21.57 (13.30-26.17)	0.285	0.429	0.120	0.440
**Tumour** max diameter (mm)	37.24 ± 18.49	30.44 ± 13.76	36.32 ± 15.75	**0.005**	**0.018**	0.990	**0.001**
**Tumour** min diameter (mm)	25.16 ± 11.88	20.45 ± 8.04	23.21 ± 9.58	**0.012**	**0.007**	0.400	**0.013**
**SUVmax**	7.29 (5.61-11.42)	9.46 (6.32-13.25)	11.15 (7.06-15.33)	**0.026**	0.343	**0.009**	0.109
**SUVmean**	3.20 (2.16-4.01)	3.68 (2.54-4.62)	4.01 (3.07-5.31)	**0.019**	0.503	**0.010**	0.073
**SUVpeak**	6.11 (3.85-8.52)	6.92 (4.73-10.57)	8.27 (6.05-12.34)	**0.023**	0.580	**0.010**	0.076
**MTV**	16.92 (7.16-49.34)	11.20 (6.32-19.96)	15.28 (7.86-29.86)	0.053	0.085	0.646	**0.037**
**TLG**	47.99 (19.43-175.96)	34.74 (18.10-84.12)	52.49 (22.12-142.48)	0.095	0.610	0.823	**0.031**

HER2, human epidermal receptor 2; SUVmax, maximum standardized uptake value; SUVmean, mean standardized uptake value; SUVpeak, peak standardized uptake value; MTV, metabolic tumour volume; TLG, total lesion glycolysis; p^1^, HER2-zero group vs. HER2-low group; p^2^, HER2-zero group vs. HER2-positive group; p^3^, HER2-low group vs. HER2-positive group.

The bold values indicate that the P-values are less than 0.05, which means they are statistically significant.

A further comparison of the clinicopathological features, PET/CT parameters and HER2 status of breast cancer patients from the two centres was conducted (**Table 3**). The CEA, CA125, CA153, tumour max diameter, SUVmax, SUVmean, Ki67 index and distant metastasis status were significantly different between patients from the two centres (all P < 0.05). The patients from Centre 2 presented higher CEA, CA125, and CA153 levels; tumour max diameters; and SUVmax, SUVmean, and Ki67 index values; and these patients exhibited a greater rate of distant metastasis. However, no significant differences were observed between the patients from Centre 1 and Centre 2 regarding age, CTmax, CTmean, short tumour diameter, SUVpeak, MTV, TLG, menopausal status, lymph node metastasis, ER status, PR status or HER2 status (all P > 0.05).

**Table 3:** Comparison of breast cancer patients between the two centres.

Variables	Centre 1 (n=157)	Centre 2 (n=84)	P value
**Age**	57.00 (49.00-64.00)	57.00 (48.00-67.00)	0.608
**CEA (ng/ml)**	1.81 (1.25-3.13)	2.49 (1.36-5.83)	**0.013**
**CA125 (ng/ml)**	11.36 (7.94-18.59)	14.41 (10.29-23.18)	**<0.001**
**CA153 (ng/ml)**	11.55 (8.39-18.28)	18.34 (9.41-34.80)	**<0.001**
**CTmax**	72.00 (65.00-81.00)	73.00 (65.00-89.00)	0.426
**CTmin**	20.69 (12.42-26.62)	19.71 (9.10-24.59)	0.140
**Tumour** max diameter (mm)	31.75 ± 13.38	37.93 ± 18.61	**0.013**
**Tumour** min diameter (mm)	21.39 ± 7.55	24.06 ± 12.22	0.403
**SUVmax**	8.81 (5.99-12.91)	11.37 (6.92-17.20)	**0.002**
**SUVmean**	3.50 (2.54-4.38)	4.15 (2.96-5.56)	**0.010**
**SUVpeak**	7.10 (4.74-10.45)	8.14 (5.29-12.89)	0.100
**MTV**	13.39 (7.27-25.88)	13.39 (4.96-35.52)	0.877
**TLG**	42.52 (22.05-103.25)	49.23 (18.79-175.60)	0.488
Menopause			0.300
Negative	94 (59.87%)	56 (66.67%)	
Positive	63 (40.13%)	28 (33.33%)	
Lymph Node Metastasis			0.706
Negative	37 (23.57%)	18 (21.43%)	
Positive	120 (76.43%)	66 (78.57%)	
Distant Metastasis			<0.001
Negative	138 (87.90%)	59 (70.24%)	
Positive	19 (12.10%)	25 (29.76%)	
ER status			0.111
Negative	63 (40.13%)	25 (29.76%)	
Positive	94 (59.87%)	59 (70.24%)	
PR status			0.115
Negative	84 (53.50%)	36 (42.86%)	
Positive	73 (46.50%)	48 (57.14%)	
Ki67 index			0.004
Negative	77 (49.04%)	25 (29.76%)	
Positive	80 (50.96%)	59 (70.24%)	
HER2 status			0.652
Zero	27 (17.20%)	12 (14.29%)	
Low	70 (44.59%)	35 (41.67%)	
Positive	60 (38.22%)	37 (44.05%)	

HER2, human epidermal receptor 2; ER, oestrogen receptor; PR, progesterone receptor; SUVmax, maximum standardized uptake value; SUVmean, mean standardized uptake value; SUVpeak, peak standardized uptake value; MTV, metabolic tumour volume; TLG, total lesion glycolysis.

The bold values indicate that the P-values are less than 0.05, which means they are statistically significant.

### 3.2 Task 1: Differentiating HER2-Zero Expression from HER2-Low/Positive Expression

RFE identified 8 features that could be used to distinguish HER2-zero expression from HER2-low/positive expression, including 3 clinical features (age, CA125, CA153), 2 CT features (CTmean and tumour min diameter) and 3 PET metabolic features (SUVmax, SUVmean, and SUVpeak). Pathological features were not included in the models.

**Figure 2:**
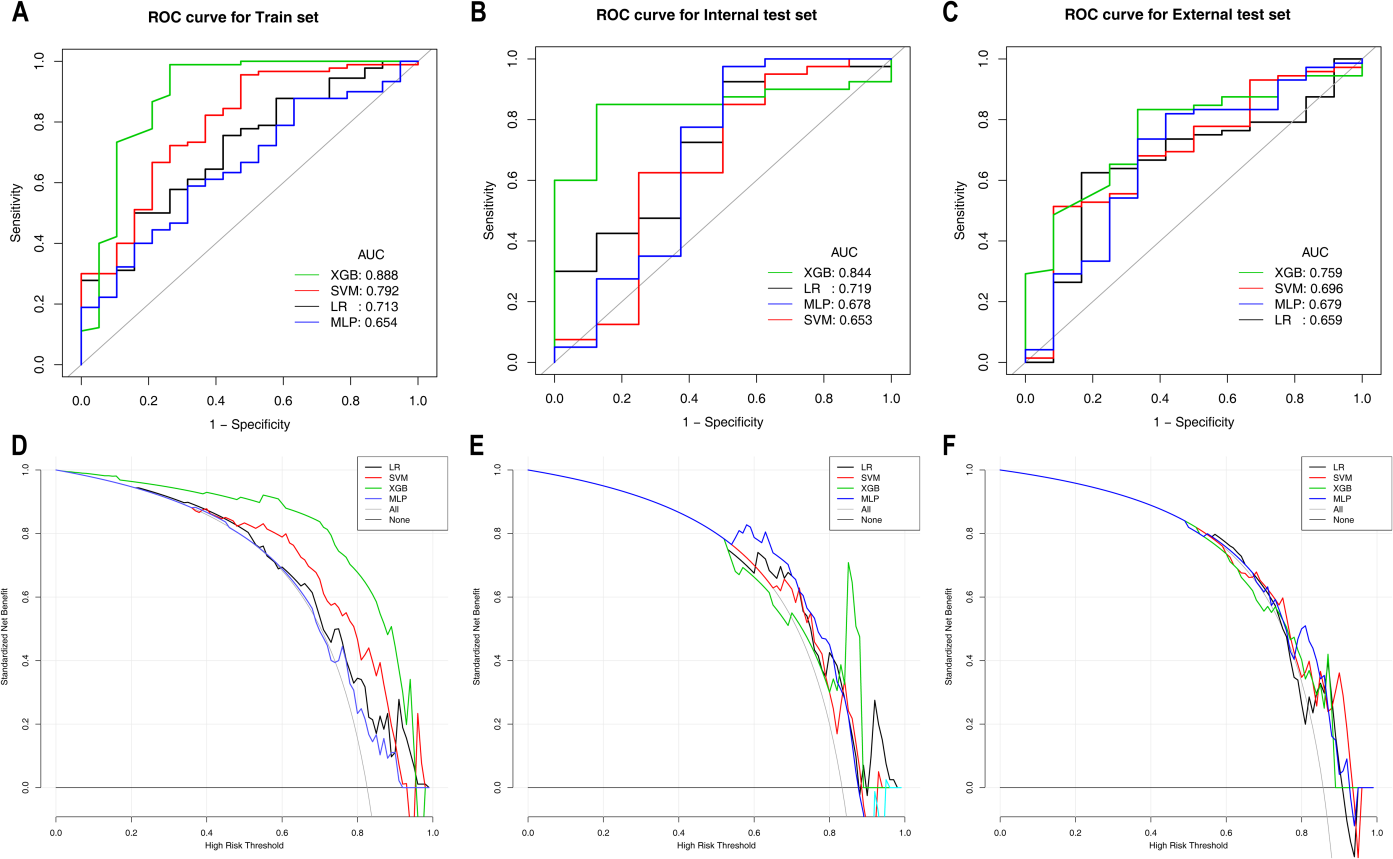
ROC and DCA curves of the machine learning models for Task 1 in the training set **(A, D)**, internal test set **(B, E)**, and external test set **(C, F)**. ROC curves are shown in **(A–C)**; DCA curves are shown in **(D–F)**. ROC, receiver operating characteristic; AUC, area under the curve; CI, confidence interval; PPV, positive predictive value; NPV, negative predictive value; LR, logistic regression; SVM, support vector machine; XGBoost, extreme gradient boosting; MLP, multilayer perceptron.

**Table 4** presents the predictive performance of ML models for differentiating HER2-zero expression from HER2-low/positive expression on the basis of the optimal feature set. In the training set, the XGBoost model not only achieved the highest AUC of 0.888 but also attained the best PPV and NPV. This model significantly outperformed both the LR model (AUC: 0.713) and the MLP model (AUC: 0.654), with DeLong test p values of 0.027 and 0.020, respectively. Moreover, the clinical benefit of the XGBoost model was significantly greater than that of the other three models. In the internal test set, the XGBoost model maintained its superior predictive performance, with the highest specificity, accuracy and PPV, achieving an AUC of 0.844. In the external test set, the XGBoost model yielded the highest sensitivity, accuracy and NPV, with an AUC of 0.759. Although the differences in the internal and external test sets were not statistically significant (DeLong test p values > 0.05), the XGBoost model demonstrated greater clinical benefit than the other models, particularly within the probability threshold range of 0.8–0.9 in the internal test set. The ROC curves and DCA for the training, internal test and external test sets are shown in **Figure 2**.

**Table 4:** Predictive performance of machine learning models for Task 1.

Model	AUC (95% CI)	Specificity	Sensitivity	Accuracy	PPV	NPV
Train set						
LR	0.713 (0.590~0.836)	0.842	0.500	0.560	0.938	0.262
SVM	0.792 (0.674~0.910)	0.526	0.956	0.881	0.905	0.714
XGBoost	0.888 (0.779~0.997)	0.737	0.989	0.945	0.947	0.933
MLP	0.654 (0.525~0.783)	0.684	0.589	0.606	0.898	0.260
Internal test set						
LR	0.719 (0.510~0.927)	0.500	0.925	0.854	0.902	0.571
SVM	0.653 (0.388~0.918)	0.750	0.625	0.646	0.926	0.286
XGBoost	0.844 (0.726~0.962)	0.875	0.850	0.854	0.971	0.539
MLP	0.678 (0.403~0.954)	0.500	0.975	0.896	0.907	0.800
External test set						
LR	0.659 (0.489~0.828)	0.833	0.625	0.655	0.957	0.270
SVM	0.696 (0.528~0.863)	0.917	0.514	0.571	0.974	0.239
XGBoost	0.759 (0.625~0.893)	0.667	0.833	0.810	0.938	0.400
MLP	0.679 (0.497~0.862)	0.583	0.819	0.786	0.922	0.350

AUC, area under the curve; CI, confidence interval; PPV, positive predictive value; NPV, negative predictive value; LR, logistic regression; SVM, support vector machine; XGBoost, extreme gradient boosting; MLP, multilayer perceptron.

### 3.3 Task 2: Differentiating HER2-low expression from HER2-positive expression

A total of 9 features were selected to differentiate between HER2-low expression and HER2-positive expression, including 2 clinical features (CEA, CA153), 1 pathological feature (PR), 1 CT feature (MaxDiam) and 5 PET metabolic features (SUVmax, MTV, TLG, SUVmean, and SUVpeak).

**Figure 3:**
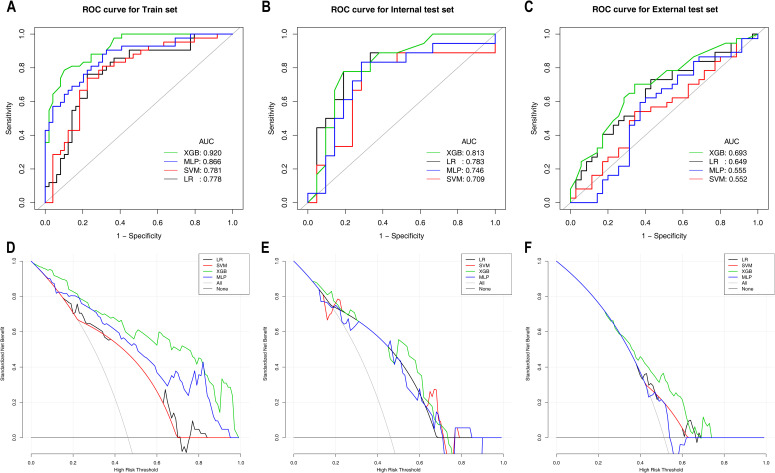
ROC curves and DCA curves of the machine learning models for Task 2 in the training set **(A, D)**, internal test set **(B, E)**, and external test set **(C, F)**. ROC curves are shown in **(A–C)**; DCA curves are shown in **(D–F)**. ROC, receiver operating characteristic; AUC, area under the curve; CI, confidence interval; PPV, positive predictive value; NPV, negative predictive value; LR, logistic regression; SVM, support vector machine; XGBoost, extreme gradient boosting; MLP, multilayer perceptron.

**Table 5** shows the diagnostic performance of ML models for differentiating HER2-low expression from HER2-positive expression. In the training set, the XGBoost model not only achieved the highest AUC of 0.920 but also attained the highest specificity, accuracy and PPV. The XGBoost model significantly outperformed the LR model (AUC: 0.778) and the SVM model (AUC: 0.781), with DeLong test p values < 0.001. The clinical benefit of the XGBoost model was also significantly greater than that of the other three models. In the internal test set, the XGBoost model maintained superior performance, with the highest specificity, accuracy and PPV, achieving an AUC of 0.814, although the difference was not statistically significant (DeLong test p values > 0.05). Additionally, within the probability threshold ranges of 0.1–0.3 and 0.5–0.65, the XGBoost model demonstrated greater clinical benefit than the other models. In the external test set, the XGBoost model achieved an AUC of 0.693 and yielded the highest specificity, accuracy, PPV and NPV, significantly outperforming the MLP model (AUC: 0.555) and the SVM model (AUC: 0.552), with DeLong test p values of 0.001 and 0.008, respectively. The clinical benefit of the XGBoost model was the greatest, as shown in **Figure 3**. The ROC curves and DCA for these sets are shown in **Figure 3**.

**Table 5:** Predictive performance of machine learning models for Task 2.

Model	AUC (95% CI)	Specificity	Sensitivity	Accuracy	PPV	NPV
Train set						
LR	0.778 (0.680~0.876)	0.776	0.762	0.769	0.744	0.792
SVM	0.781 (0.684~0.878)	0.776	0.738	0.758	0.738	0.776
XGBoost	0.920 (0.868~0.972)	0.898	0.786	0.846	0.868	0.830
MLP	0.866 (0.792~0.940)	0.674	0.905	0.780	0.704	0.892
Internal test set						
LR	0.783 (0.617~0.949)	0.810	0.778	0.795	0.778	0.810
SVM	0.709 (0.531~0.887)	0.667	0.833	0.744	0.682	0.824
XGBoost	0.814 (0.673~0.954)	0.810	0.778	0.795	0.778	0.810
MLP	0.746 (0.580~0.913)	0.714	0.833	0.769	0.714	0.833
External test set						
LR	0.649 (0.521~0.778)	0.571	0.730	0.653	0.643	0.667
SVM	0.552 (0.417~0.687)	0.657	0.541	0.597	0.625	0.575
XGBoost	0.693 (0.571~0.816)	0.657	0.703	0.681	0.684	0.677
MLP	0.555 (0.416~0.694)	0.629	0.595	0.611	0.629	0.595

AUC, area under the curve; CI, confidence interval; PPV, positive predictive value; NPV, negative predictive value; LR, logistic regression; SVM, support vector machine; XGBoost, extreme gradient boosting; MLP, multilayer perceptron.

### 3.4 SHAP algorithm for the interpretation of model decision-making processes

**Figure 4:**
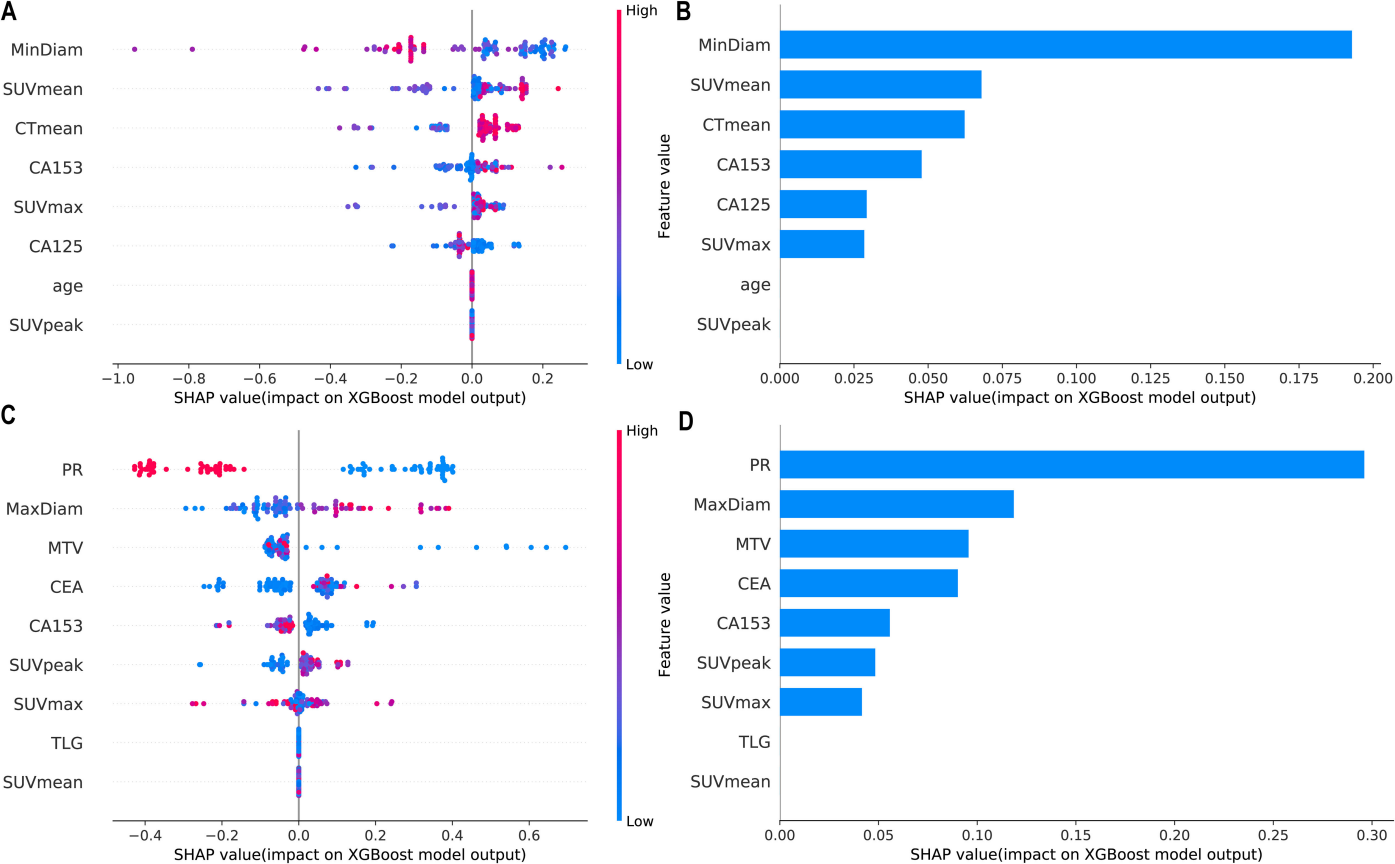
Interpretability SHAP value analysis of the XGBoost models for Task 1 **(A, B)** and Task 2 **(C, D)**. **(A, C)** Feature importance ranking based on SHAP values. The position on the Y-axis represents the importance ranking, and the X-axis reflects the association between each value of a feature and the corresponding SHAP value. **(B, D)** Importance rankings of the included features according to the mean (|SHAP value|). PR, progesterone receptor; SUVmax, maximum standardized uptake value; SUVmean, mean standardized uptake value; SUVpeak, peak standardized uptake value; MTV, metabolic tumour volume; TLG, total lesion glycolysis.

SHAP values were calculated for the features in the XGBoost models for Task 1 and Task 2. The Y-axis represents the ranking of features by importance, whereas the X-axis shows the relationship between each feature value and its corresponding SHAP value. A SHAP value greater than zero indicates a positive contribution to the outcome (**Figure 4**).

**Figure 5:**
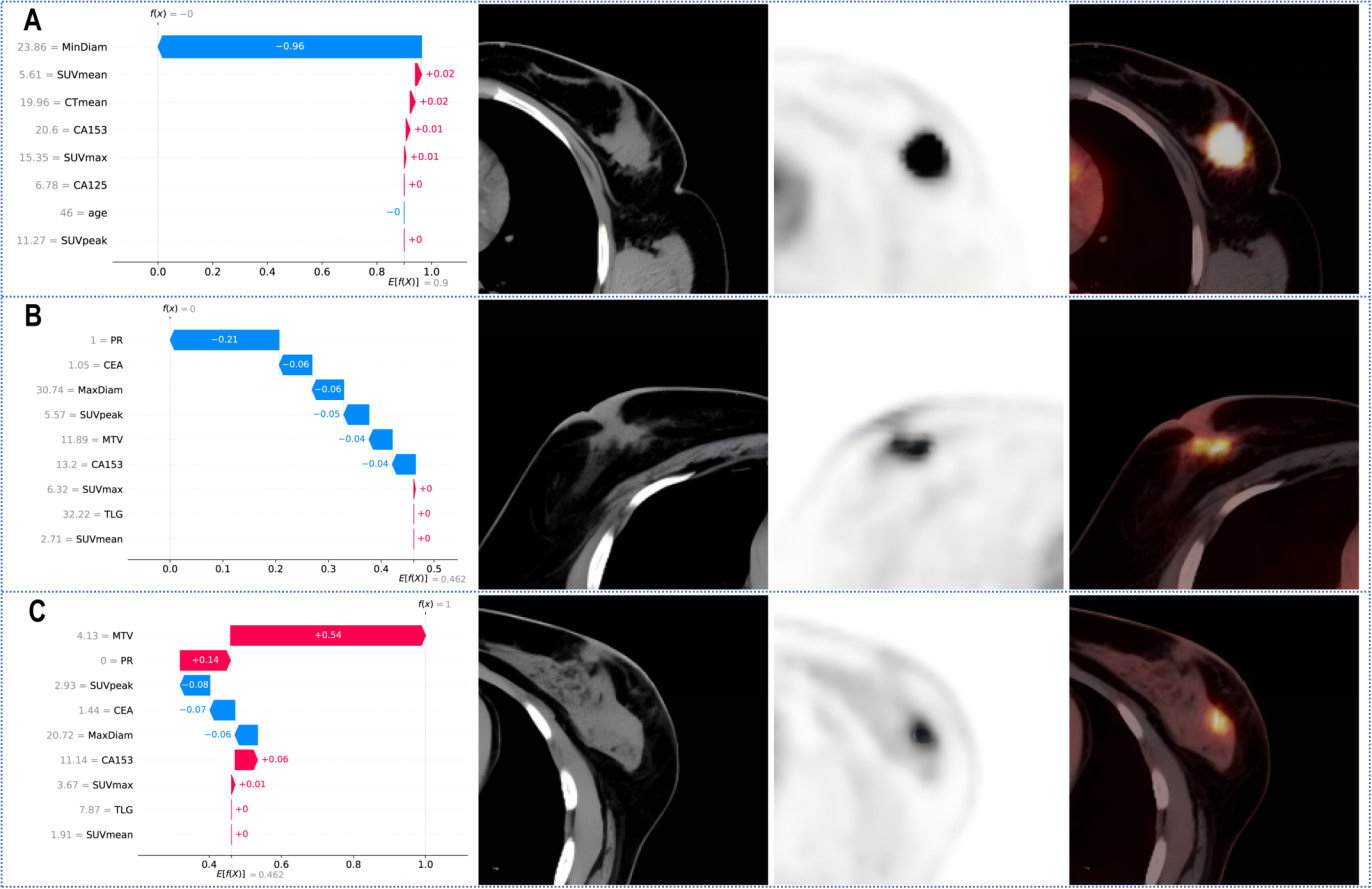
SHAP waterfall plot for predicting **(A)** HER2-zero, **(B)** HER2-low, and **(C)** HER2-positive tumours. PR, progesterone receptor; SUVmax, maximum standardized uptake value; SUVmean, mean standardized uptake value; SUVpeak, peak standardized uptake value; MTV, metabolic tumour volume; TLG, total lesion glycolysis.

**Figure 5** shows the personalized prediction plots for (A) HER2-zero, (B) HER2-low, and (C) HER2-positive tumours. SHAP values quantify the contributions of features to predictions about HER2 expression by decomposing model outputs into additive feature effects. The baseline expectation E[f(x)] represents the model’s prior probability without feature inputs, whereas f(x) reflects the adjusted probability after feature integration. Red features increase positive predictions, and blue features increase negative predictions. The arrow length and the number of the arrows represent the impact on the predictions; the longer the arrow and the larger the number are, the greater the influence on the model’s prediction. In the XGBoost model for Task 1, the three features with the highest weights are the tumour min diameter, SUVmean and CTmean, and larger tumour min diameters and lower SUVmean values are associated with HER2-zero expression. In the XGBoost model for Task 2, the top three features with the highest weights are the PR status, tumour max diameter and MTV, and a negative PR status and longer tumour max diameter are more likely to indicate HER2-positive expression.

## 4 Discussion

This study revealed that various ML models that were constructed using ^18^F-FDG PET/CT imaging parameters combined with clinicopathological features performed well in identifying the HER2 expression status of patients with breast cancer. Among these models, the XGBoost model, which showed the best predictive performance, achieved AUC values ranging from 0.693 to 0.844 in both the internal and external test sets, indicating good model robustness and providing valuable support for clinical decision-making for patients with breast cancer.

Previous studies have confirmed the correlation between ^18^F-FDG metabolic parameters and HER2 expression. Patients with HER2-positive breast cancer have higher SUVmax values than those with HER2-negative breast cancer (26, 27, 33–35). In this study, we not only confirmed that metabolic parameters such as the SUVmax and MTV are correlated with HER2 expression but also developed ML models to predict different HER2 expression statuses. Our results revealed that the SUVmax, SUVmean, and SUVpeak consistently increased across the three groups of patients with HER2-zero, HER2-low, and HER2-positive expression. As shown in **Table 2**, significant differences were found only between the HER2-zero and HER2-positive groups, indicating that HER2-positive tumours have higher metabolic indicators than HER2-zero tumours, suggesting greater invasiveness. Additionally, we observed differences in tumour size (max and min diameters) and ER/PR status across groups with different HER2 expression statuses. This finding is consistent with previous findings that HER2-positive tumours tend to be larger and more likely to be ER-/PR-negative (25, 30). Furthermore, our study uniquely leveraged SHAP analysis to provide population-level feature importance rankings and personalized prediction visualizations, thereby significantly enhancing the clinical interpretability of the decision-making process involving multiple parameters.

Furthermore, a variety of ML models were developed using ^18^F-FDG PET/CT parameters and clinicopathological features to differentiate between HER2-zero expression and HER2-low/positive expression. As shown in **Figure 2**; **Table 4**, among these models, the XGBoost model demonstrated superior predictive performance and clinical benefit. RFE identified a total of 8 features from among the clinical features and ^18^F-FDG PET/CT parameters. Notably, pathological features were not included in the model, as shown in **Figure 4**. Therefore, we can achieve noninvasive prediction of HER2-zero expression using only clinical indicators combined with ^18^F-FDG PET/CT parameters without the need for IHC results. This approach facilitates faster treatment planning and prognosis assessment and reduces the need for invasive biopsies in certain patients, improving patient comfort and lowering the risk of surgical complications. As shown in **Figures 4**, **5**, the three features with the highest weights in the XGBoost model were the tumour min diameter, SUVmean and CTmean. Moreover, as shown in **Figure 4**, a higher tumour min diameter, lower SUVmean, and lower CTmean were associated with HER2-zero expression, which has not been clearly reported in previous studies. As shown in **Table 4**, although the XGBoost model demonstrated strong overall performance in differentiating HER2 expression statuses (AUC: 0.759-0.844), its NPV in the external test set was relatively low. One potential reason for this finding is the relatively small number of HER2-zero patients in the dataset, which may have affected the model’s ability to identify these cases accurately. Future research could help improve the model’s NPV by increasing the sample size of HER2-zero patients, especially with multicentre, large-sample datasets. This would likely increase the model’s performance and clinical value. Furthermore, future studies could consider introducing more features or optimizing training methods to further improve the model’s performance, particularly with respect to improving the NPV.

As shown in **Figure 3**; **Table 5**, among the various models that distinguish between HER2-low expression and HER2-positive expression, the XGBoost model achieved the best predictive performance and clinical benefit. Among the 9 features that were used for modelling, as shown in **Figure 4**, the pathological characteristic PR status had the highest feature importance, followed by the tumour max diameter and MTV. Additionally, the personalized SHAP prediction plots in **Figure 5** show that the MTV has greater predictive value for HER2-positive breast cancer patients than for HER2-low breast cancer patients. Therefore, for cases with equivocal IHC results, the XGBoost model, which incorporates pathological features, optimizes diagnostic workflows by reducing reliance on ISH testing, thereby shortening clinical decision-making timelines and lowering healthcare costs.

Mao et al. conducted a multivariate logistic regression analysis utilizing four MRI diffusion model parameters to differentiate between HER2-low and HER2-positive breast cancer. By incorporating tumour size and ER/PR status into the model, they achieved an AUC of 0.877 (31). However, that study had a relatively small sample size, with only 158 cases. Huang et al. developed four ML models based on MRI parameters to identify HER2-zero and HER2-low breast cancer, with AUC values of 0.783 and 0.787 in the training and validation sets, respectively (32). These studies were all single-centre studies and lacked external validation. In contrast, our study is a dual-centre study and categorized the patients into three groups according to HER2 expression status (HER2-zero, HER2-low, and HER2-positive) for comparison, providing a more comprehensive analysis. Additionally, the model still demonstrated good diagnostic performance in the external validation cohort, increasing the reliability of the results. These findings provide new insights into the relationships among HER2 expression status, tumour clinicopathological features, and ^18^F-FDG PET/CT imaging parameters in breast cancer. The established prediction models may contribute to personalized treatment plans and prognosis assessment for breast cancer patients.

This study has the following limitations. First, it was a retrospective study, which may introduce bias in the inclusion of the study population, and the results are representative only of the Chinese population. Second, although this study implemented stratified sampling and class weight adjustment to mitigate data imbalance, the limited sample size of HER2-zero breast cancer patients still impacted model robustness, as evidenced by the relatively low NPV in the external validation set of the XGBoost model in Task 1. Future investigations should focus on constructing larger multicentre datasets while exploring advanced data augmentation techniques, such as integrating synthetic minority oversampling (SMOTE) with GAN-based PET/CT image generation coupled with cross-modal transfer learning frameworks, to simultaneously increase model performance in terms of class balance and imaging feature generalizability. Third, PET/CT involves nonnegligible radiation exposure, particularly in scenarios that require repetitive imaging. Future studies could explore multimodal imaging strategies (e.g., PET/MRI) to optimize the trade-off between diagnostic accuracy and radiation safety. Additionally, recent studies have shown that radiomics based on ultrasound and MRI has the potential to predict different HER2 expression statuses in patients with breast cancer, including patients with HER2-low expression (36–38). This study used ^18^F-FDG PET/CT imaging parameters and clinicopathological features rather than radiomics features, and future work will involve related radiomics research.

## 5 Conclusions

In conclusion, ML models developed on the basis of preoperative ^18^F-FDG PET/CT parameters and clinicopathological features can help distinguish different HER2 expression statuses in patients with breast cancer. Furthermore, noninvasive prediction of HER2-zero expression can be achieved solely by combining clinical indicators with PET/CT parameters. In cases where the immunohistochemistry results are ambiguous or borderline, predicting HER2-low expression using PET/CT parameters combined with clinicopathological features still has significant clinical value.

## Data Availability

The datasets used and/or analyzed during the current study are available from the author upon reasonable request. Requests to access these datasets should be directed to Jianxiong Gao, gjx970113@163.com.
